# Evaluation of the Advantages of Silicone Stent in Revision Endoscopic Dacryocystorhinostomy in a Tertiary Care Hospital

**DOI:** 10.1055/s-0046-1818565

**Published:** 2026-04-30

**Authors:** Charushila Sonawane, Ritu Radheshyam Raval, Anushree Bajaj, Bhalchandra Paike, Tanvi Patil

**Affiliations:** 1 Department of Otorhinolaryngology, Dr. Ulhas Patil Medical College & Hospital, Jalgaon, Maharashtra, India

**Keywords:** silicone stent, nasolacrimal duct obstructions, chronic dacryocystitis, epiphora

## Abstract

**Introduction:**

Silicone stents are often employed in dacryocystorhinostomy (DCR) procedures to maintain the patency of the newly created tear drainage passage during the healing stage. These stents are flexible, biocompatible, and generally well tolerated by patients. They help prevent adhesion formation and ensure that the surgical opening remains open as the tissue heals. Use of stents in primary DCR is well documented, but their advantages in revision DCR need further exploration. We evaluated the advantages of silicone stents in revision endoscopic DCR performed at a tertiary care hospital.

**Objective:**

To compare the surgical success rates of revision endoscopic DCR with and without the use of silicone stents, analyze the incidence of postoperative complications, assess patient satisfaction and quality of life following revision endoscopic DCR using standardized patient questionnaires and evaluate the effectiveness of silicone stents in maintaining ostium patency during the healing process

**Methods:**

The present prospective, interventional, hospital-based study involved 160 patients with nasolacrimal duct obstruction with patent upper and lower canaliculi with symptoms like epiphora, discharge from the eye, swelling in the lacrimal region, hard stop on lacrimal syringing and previous failed endoscopic endonasal DCR and common canalicular obstruction for 2 years.

**Results:**

When compared with the non-silicone stent group, the silicone stent group experienced shorter hospital stays, larger anatomical patency, lower recurrence rates, higher satisfaction, and higher success rates.

**Conclusion:**

In revision DCR, the use of silicone stents greatly improves surgical results, patient satisfaction, recurrence rates, and hospital stays.

## Introduction

Nasolacrimal duct blockages can be treated using endoscopic dacryocystorhinostomy (DCR), a minimally-invasive surgical technique. This condition often results in chronic dacryocystitis, epiphora (excessive tearing), and recurrent eye infections, significantly impacting patients' quality of life. A skin incision is required for traditional external DCR, which may result in scarring and require longer time to heal. In contrast, endoscopic DCR, performed through the nasal cavity using endoscopic techniques, avoids external scars, reduces postoperative discomfort, and generally allows for quicker recovery. 1

While primary endoscopic DCR has a high success rate, failures do occur, requiring revision surgery. The reasons for failure can include inadequate ostium size, granulation tissue formation, scarring, and improper healing, leading to recurrent obstruction. Revision endoscopic DCR is technically more challenging due to altered anatomy from previous surgery, scar tissue, and potentially more complex causes of obstruction. 2

Silicone stents are often employed in DCR procedures to maintain the patency of the newly created tear drainage passage during the healing process. These stents are flexible, biocompatible, and generally well tolerated by patients. They help prevent adhesion formation and ensure that the surgical opening remains open as the tissue heals. The use of stents in primary DCR is well documented, but their advantages in revision DCR need further exploration. 3 4

Primary endoscopic DCR boasts success rates ranging from 85 to 95%. However, because of recurring blockages or chronic discomfort, some individuals need revision surgery. The success of primary DCR depends on various factors, including the surgeon's experience, patient anatomy, and the underlying cause of the obstruction. 5 6

It is anticipated that the use of silicone stents will demonstrate significant advantages in revision endoscopic DCR, leading to higher success rates, fewer complications, and improved patient satisfaction. The findings from our study could inform clinical practice guidelines, helping to standardize the use of stents in revision DCR and ultimately enhancing patient care. 7

The evaluation of silicone stents in revision endoscopic DCR represents a critical area of research with the potential to improve surgical outcomes and patient's quality of life. By systematically analyzing the benefits of silicone stents in a controlled clinical setting, our study aims to provide evidence to guide clinical decision-making and optimize the management of nasolacrimal duct obstructions in tertiary care hospitals. 8

The primary objective of our study is to evaluate the advantages of silicone stents in revision endoscopic DCR performed at a tertiary care hospital. This evaluation will include an analysis of surgical outcomes, complication rates, patient satisfaction, and overall success rates of the procedures. Understanding these advantages is important for refining surgical techniques, improving patient outcomes, and developing standardized protocols for managing nasolacrimal duct obstructions. 9

## Methods

The present prospective, interventional, hospital-based study was performed in the Department of Ophthalmology and Outpatient Department of otorhinolaryngology of a tertiary care institute over a period of 2 years (October 2022 to September 2024). The study enrolled patients ≥ 15 years, of either sex, with nasolacrimal duct obstruction with patent upper and lower canaliculi, symptoms like epiphora, discharge from the eye, swelling in the lacrimal region, hard stop on lacrimal syringing and previous failed endoscopic endonasal DCR, common canalicular obstruction, and subjects giving consent for research. While patients with of congenital dacryocystitis, gross systemic diseases, chronic granulomatous diseases of the nose, polyps and subjects not giving consent were excluded.

The study commenced after obtaining approval of the Institutional Ethics Committee and written informed consent of the patients. Based on the treatment received, a total of 160 patients were selected for the study. Written informed consent was taken; preoperative assessment of patients which includes detailed history of the patient, detailed ENT examination of patient was done.

Classification of epiphora based on examination was as follows: a) Stage 0–no epiphora; b) Stage 1–minimal epiphora; c) Stage 2–moderate epiphora; d) Stage 3–severe epiphora.

Revision DCR with and without silicone stenting was done. Postoperative assessment of patients done at 1, 3, and 6 months and 1 year, which included grading of success, with grade 0, 1, and 2, being classified as success and grade 3 as complete failure. Anatomical patency was assessed by lacrimal syringing under endoscopic guidance as patent or not patent. Assessment of recurrence was made postoperatively in follow-up visits.

## Statistical Analyses

The data was analyzed with IBM SPSS Statistics for Windows (IBM Corp.) software, version 23.0.

Minimum sample size (n)—to test the equality of two proportions:



where:

p1 = 0.08 q1 = 0.92
p2 = 0.22 q2 = 0.78
*p* = 0.15 q = 0.85
Z1 = 1.64 at α = 5% level of significance
Z2 = 0.84 at 80% power of test.

*n* = 80

∴ Minimum sample size for the study will be 80 in each group

*n*_1_ = 80 and *n*_2_ = 80

## Results

There was a marginally greater proportion of women in both categories. The distribution varied across age groups but did not show a consistent pattern favoring one group over the other, with mean age of 46 years. There was a minor difference in symptom duration between the two groups, with the no-silicone stent group reporting a slightly longer duration on average. There were no significant differences observed in the distribution of these comorbidities between the 2 groups ( *p* -value of 0.831).

Similar distributions were seen between the silicone stent and no silicone stent groups when lacrimal drainage system blockages were staged. Stage 2 (moderate) obstructions were the most common in both groups, accounting for 43.75% of cases in the silicone stent group and 42.5% in the group without a silicone stent. While stage 1 (minimum) obstructions were reported by 25% and 27.5% of the silicone stent and no silicone stent groups, respectively, stage 3 (severe) obstructions were rather less common, accounting for 31.25% of the silicone stent group and 30% of the no silicone stent group.

The comparison of hospital stays between the silicone stent and no-silicone stent groups revealed a significant difference, with a mean stay of 3 days for the silicone stent group and 4 days for the no-silicone stent group, resulting in an overall mean stay of 3.5 days across both groups ( *p* = 0.042). This suggests that patients with silicone stents tended to have shorter hospital stays compared with those without silicone stents ( Table 1 ).

**Table 1:** Demographic and clinical characteristics

Characteristics	Silicone-stent group (n = 80)	No silicone stent group (n = 80)	Total (n = 160)	
**Sex: n (%)**				
Male	35	33	68	
Female	45	47	92	
**Age-wise patient distribution (years)**				
< 25	10	11	21	
26–35	16	15	31	
36–45	19	18	38	
46–55	18	20	38	
56–65	10	11	21	
≥ 66	7	5	12	
**Mean age (years** )	45	47	46	
**Duration of symptoms (months)**	12	13	12.5	
**Socioeconomic status: n (%)**				*p* -value
Low	30 (37.5%)	35 (43.75%)	65 (40.63%)	
Middle	40 (50%)	35 (43.75%)	75 (46.88%)	
High	10 (12.5%)	10 (12.5%)	20 (12.5%)	0.765
**Type of residence: n (%)**				
Urban	45 (56.25%)	50 (62.5%)	95 (59.38%)	
Rural	35 (43.75%)	30 (37.5%)	65 (40.63%)	0.451
**Comorbidities:** **n (%)**				
Diabetes	20 (25%)	18 (22.5%)	38 (23.75%)	
Hypertension	15 (18.75%)	17 (21.25%)	32 (20%)	
Both	10 (12.5%)	8 (10%)	18 (11.25%)	
None	35 (43.75%)	37 (46.25%)	72 (45%)	
**Preoperative symptoms and classification: n (%)**				
Epiphora	80 (100%)	80 (100%)	160 (100%)	
Discharge from the eye	60 (75%)	62 (77.5%)	122 (76.25%)	
Swelling in lacrimal region	30 (37.5%)	32 (40%)	62 (38.75%)	
**Classification of epiphora: n (%)**				
Stage 0 (no epiphora)	0	0	0	
Stage 1 (minimal)	20 (25%)	22 (27.5%)	42 (26.25%)	
Stage 2 (moderate)	35 (43.75%)	34 (42.5%)	69 (43.12%)	
Stage 3 (severe)	25 (31.25%)	24 (30%)	49 (30.63%)	
**Patient hospital stay (days)**				
Mean stay	3	4	3.5	0.042

In the silicone-stent group, higher satisfaction was noted (75%) compared with 67% in the no silicone-stent group. Notably, a smaller proportion of patients in the silicone-stent group were not satisfied compared with the no silicone -stent group. These results indicate higher overall patient satisfaction in the silicone-stent group ( Table 2 ).

**Table 2:** Patient satisfaction

Satisfaction level: n (%)	Silicone-stent group (n = 80)	No silicone-stent group (n = 80)
**Very satisfied**	60 (75%)	54 (67%)
**Satisfied**	15 (18.75%)	15 (31.25%)
**Not satisfied**	5 (6.25%)	14 (8%)

**Fig. 1:**
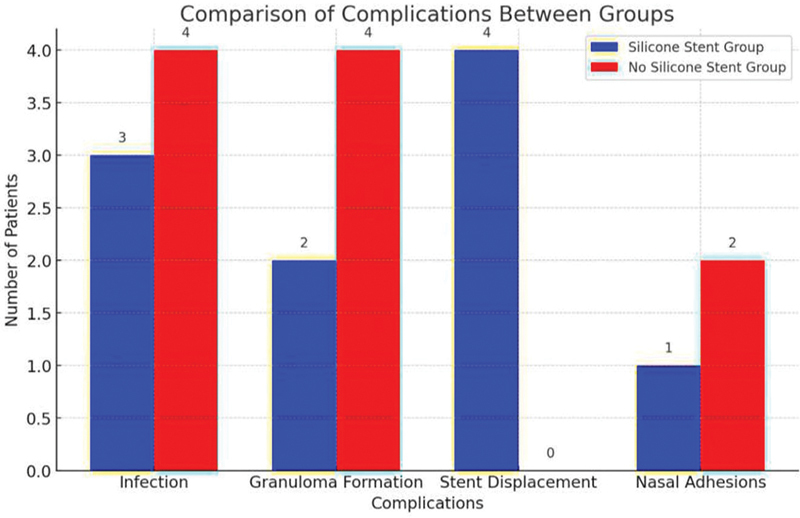
Incidence of complications among the study sample.

Infection rates, granuloma formation, and nasal adhesions were slightly lower in the silicone-stent group. Stent displacement occurred in 5% in the silicone-stent group. These findings suggest that the silicone-stent group had a lower incidence of certain complications ( Fig. 1 ).

**Fig. 2:**
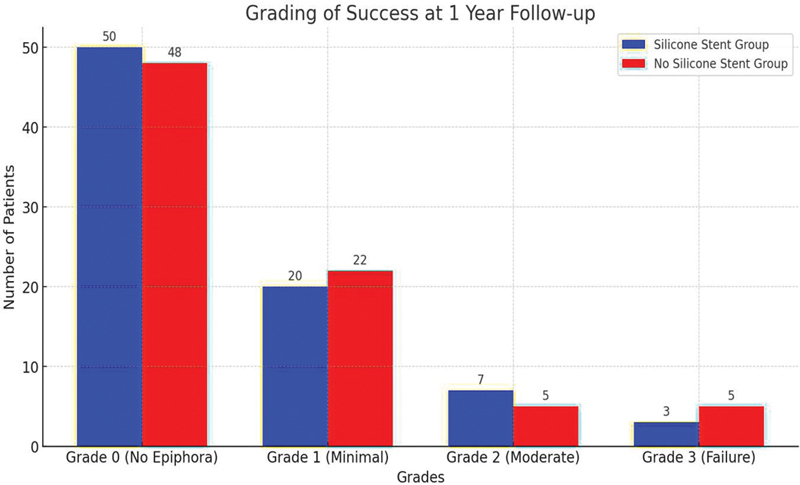
Grading of success at the 1-year follow-up.

The total success rate, encompassing grades 0 to 2, was higher in the silicone-stent group (96.25%) compared with the no silicone-stent group, with a significant *p* -value of < 0.047. These results highlight a higher overall success rate in the silicone-stent group ( Table 3 and Fig. 2 ).

**Table 3:** Grading of success (at 1-year follow-up)

Grade: n (%)	Silicone-stent group (n = 80)	No silicone-stent group (n = 80)	*p* -value
Grade 0 (no epiphora)	50 (62.5%)	48 (60.5%)	
Grade 1 (minimal)	20 (25%)	22 (26.25%)	
Grade 2 (moderate)	7 (8.75%)	5 (10.5%)	
Grade 3 (failure)	3 (3.75%)	5 (6.25%)	< 0.033
Total success (grades 0–2)	77 (96.25%)	75 (92.25%)	< 0.047

According to the results, the silicone-stent group surpassed the no-silicone-stent group in terms of anatomical patency at the 1-year follow-up ( Table 4 ).

**Table 4:** Anatomical patency (at 1-year follow-up)

Anatomical patency: n (%)	Silicone-stent group (n = 80)	No silicon-stent group (n = 80)	*p* -value
**Patent**	77 (96.25%)	74 (92.75%)	< 0.041
**Not patent**	3 (3.75%)	6 (5.25%)	< 0.018

These findings point to the achievable advantage of silicone stents in lowering recurrence, suggesting a somewhat reduced recurrence rate in the silicone-stent group with a significant *p* -value ( Table 5 ).

**Table 5:** Recurrence rate (at 1-year follow-up)

Recurrence: n (%)	Silicone-stent group (n = 80)	No silicone-stent group (n = 80)	*p* -value
**Recurrence**	5 (6.25%)	7 (8.5%)	0.040
**No recurrence**	75 (93.75%)	73 (92.5%)	0.039

## Discussion

Endoscopic DCR is a well-established surgical procedure for treating nasolacrimal duct obstruction. However, the effectiveness of revision DCR, particularly with the use of silicone stents, remains a subject of considerable interest and debate within the medical community.

In our study, the distribution of patients across different age groups revealed a varied pattern without a consistent trend favoring either group. Specifically, the silicone-stent group had slightly higher numbers in the < 25, 26 to 35, and 36 to 45 years of age brackets, while the no silicone-stent group had marginally more cases in the 46-to-55 and 56-to-65 years of age brackets. The variability suggests that age distribution alone does not significantly influence the decision to use silicone stents, highlighting the need for a more nuanced understanding of patient-specific factors that may drive the choice of surgical intervention.

Both groups were relatively similar in age distribution, indicating that the benefits and risks of silicone stent placement were considered across a similar age range of patients, ensuring a balanced comparison.

The study's gender distribution revealed a slightly higher proportion of women, which is consistent with previous research that suggests a higher prevalence of nasolacrimal duct obstruction in women, maybe as a result of anatomical and hormonal variations. The slight differences in gender distribution between the groups were not significant enough to impact the overall outcomes of the study. Socioeconomic factors did not play a major role in determining the type of surgical intervention neither did the type of residence, which indicates the widespread applicability of silicone stents in diverse residential settings. The comorbid conditions did not disproportionately affect the outcomes of the two groups. The similar distribution of comorbidities ensures that the comparative analysis remains unbiased by underlying health conditions, thereby strengthening the validity of the study's conclusions regarding the efficacy of silicone stents. The slightly longer duration in the no silicone-stent group might reflect a delay in seeking revision surgery or differences in initial treatment responses.

The staging of lacrimal drainage system obstructions was similar between the two groups. Stage 2 (moderate) obstructions were the most prevalent. Stage 3 (severe) obstructions were slightly less common.

The mean hospital stay was significantly shorter in the silicone-stent group, that is, 3 days, which suggests that the use of silicone stents may facilitate faster recovery and discharge, potentially reducing hospital resource utilization and associated costs. The shorter hospital stay could be attributed to the mechanical support provided by the stents, promoting quicker postoperative stabilization.

Compared with 67% in the group without silicone stents, 75% of those in the silicone-stent group had high levels of satisfaction, indicating higher overall patient satisfaction in this group.

According to these results, the silicone-stent group experienced fewer problems, such as decreased rates of infection, granuloma formation, and nasal adhesions. Stent displacement was seen in the stent group.

Greater overall success rate was observed for grades 0-to-2 epiphora in the silicone-stent group. In comparison to the no silicone-stent group, the silicone-stent group exhibited a greater rate of anatomical patency at the 1-year follow-up (96.25%).

The results suggest somewhat reduced recurrence rate in the silicone-stent group, suggesting that silicone stents could prove useful in lowering recurrence with significant *p* -value.

The overall success rate was higher in the silicone-stent group in our study, similar to Dutta et al., while the other studies reported no significant difference 10 11 12 ( Table 6 ).

**Table 6:** Comparison of our study's results with those of other studies

Study	Our study (2024)	Kim et al. 10 (2018)	Kang et al. 11 (2018)	Dutta et al. 12 (2023)
**Sample size**	160	587	1,216	40
**Mean age**	46	Not specified	42	Not specified
**Gender distribution**	68 male patients, 92 female patients	Not specified	Male predominance	Not specified
**Duration of symptoms (months)**	Silicone stent: 12, no silicone stent: 13	Not specified	Not specified	Not specified
**Socioeconomic status**	No significant difference ( *p* = 0.765)	Not specified	Not specified	No significant difference
**Type of residence**	Urban: 56.25% (silicone), 62.5% (no silicone)	Not specified	Not specified	Not specified
**Comorbidities**	No significant difference ( *p* = 0.831)	Not specified	No significant difference	No significant difference
**Preoperative symptoms**	100% epiphora, 75% discharge (silicone), 77.5% discharge (no silicone)	Not specified	Not specified	100% epiphora
**Classification of epiphora**	Stage 2: 43.75% (Silicone), 42.5% (no silicon)	Not specified	Not specified	Not specified
**Patient hospital stay (days)**	Silicone stent: 3, no silicone stent: 4 ( *p* = 0.042)	Not specified	Not specified	Not specified
**Complications**	Infection: 3.75% (silicone), 5% (no silicone); Granuloma: 2.5% (silicone), 4.50% (no silicone)	No significant difference	Infection: 4.22%	Granulations: 50% (silicone), 25% (no silicone)
**Grading of success**	96% success in stenting, 91% without stenting	No significant difference	No significant difference	95% success in stenting, 85% without stenting
**Overall success rate**	Higher in silicone group	No significant difference	No significant difference	85% (no stent), 95% (stent)

The current research not only adds to the growing body of evidence but also provides valuable insights into the demographic factors, clinical presentations, and postoperative outcomes associated with the use of silicone stents in DCR.

One of the primary reasons the present study is important is its potential to enhance clinical outcomes for patients with nasolacrimal duct obstruction (NLDO),as it underscores the effectiveness of silicone stents in maintaining long-term duct patency and reducing the likelihood of recurrent obstruction, which is crucial for patient quality of life and satisfaction.

Dacryocystorhinostomy may be conducted either externally (EXT-DCR) or endoscopically (EN-DCR). External DCR has been considered the “gold standard.” However, because recent studies revealed the efficacy and safety of EN-DCR in identifying postnasal obstruction of the nasolacrimal pathway, this technique has become increasingly popular. Overall, the outcomes after EN-DCR and EXT-DCR are comparable, with good results maintained over time.

The study also highlighted the economic benefits associated with the use of silicone stents. Patients in the silicone-stent group had a significantly shorter hospital stay, which could lead to more efficient utilization of hospital resources and reduction in the burden on healthcare facilities.

Patient satisfaction is a critical outcome measure in any surgical procedure, and this study demonstrated significantly higher satisfaction rates in the silicone-stent group. This higher satisfaction rate can be attributed to the improved symptom relief, shorter recovery time, and lower complication rates associated with silicone stent placement.

The results of the current study have the potential to inform clinical practice guidelines and decision-making processes in the management of NLDO. The significant improvements in clinical outcomes associated with silicon-stent placement can guide ophthalmologists and ENT specialists in recommending the most effective surgical interventions for their patients. Incorporating silicone-stent placement into standard practice for revision DCR procedures can lead to more consistent and favorable patient outcomes.

## Conclusion

Our study concludes that silicone-stent placement in revision endoscopic DCR significantly enhances surgical outcomes in a tertiary care hospital setting. Compared with patients without silicone stents, those who had them showed better anatomical patency, lower recurrence rates, shorter hospital stays, and higher levels of satisfaction. The use of stents in revision cases prevents the need of regular syringing for 3 months to keep neo-ostium patent. These findings underscore the efficacy of silicone stents in managing nasolacrimal duct obstructions, offering a reliable intervention to improve patient quality of life and surgical success rates. The results support the routine incorporation of silicone stents in revision DCR procedures, providing a valuable guideline for optimizing patient care and treatment outcomes.
